# Effects of Nutritional Interventions on Intestinal Microbiota

**DOI:** 10.3390/nu15122694

**Published:** 2023-06-09

**Authors:** Marta Stelmach-Mardas

**Affiliations:** Department of Obesity Treatment, Metabolic Disorders and Clinical Dietetics, Poznan University of Medical Sciences, 60-569 Poznan, Poland; stelmach@ump.edu.pl

The gut microbiota is considered a new probable factor strongly connected with pathogenesis of many civilization’s diseases. Currently, proper diet modification, including probiotic supplementation, is the key challenge as both may reduce the clinical symptoms of the diseases or influence the treatment outcome. Commensal microbiota plays a crucial role in the regulation of intestinal immune homeostasis. The bacteria in the family *Lactobacillus* are the most frequently studied in the field of oncology/gastroenterology. These are possibly related to the reduction in undesirable effects such as: diarrhea, constipation, nausea and vomiting. In this Special Issue, several important points were addressed to open the space for further discussion on microbiota and the use of functional food supplementation.

One of them introduced “Bryndza” cheese as a functional food rich in lactic acid bacteria. Hric et al. [1] showed that the short-term weight loss program based on “Bryndza” cheeses consumption improves body composition and increases the abundance of: *Lactobacillales*, *Streptococcaceae*, *Lactococcus* and *Streptococcus* and short-chain fatty acids (SCFAs) producers such as: *Phascolarctobacterium* and *Butyricimonas*. Another study by Konstanti et al. [2] focused on bovine lactoferrin (bLF) and its immunomodulatory properties. Authors proved that bLF enhanced the relative abundance of *Holdemanella* in the fecal microbiota of healthy elderly women, and the further addition of active galactooligosaccharides (GOS) enhanced the relative abundance of *Bifidobacterium.* Additionally, concentrations of SCFAs, calprotectin, zonulin and alpha-1-antitrypsin did not change during this dietary intervention. The positive effects on gut microbiota, SCFAs (higher proportion of propionic acid and lower proportion of acetic acid) and atherosclerotic plaque amount were observed in the study carried out by Liu et al. [3]. Specifically, the diet based on whole beans and the isolated fiber fraction resulted in a lower *Firmicutes*/*Bacteroidetes* ratio, a higher relative abundance of unclassified S24-7, *Prevotella*, *Bifidobacterium*, and unclassified *Clostridiales* and a lower abundance of *Lactobacillus.* The study presented by Jin et al. [4] provides a basis for the development of ellagic acid (EA)-rich functional food supplemented with W. coagulans BC2000 as the combined effect of both. This activated the autophagy pathway in the mouse liver; a urolithin-like effect, with altered β-diversity of gut microbiota and increased *Eggerthellaceae*, was observed. In the systematic review, the influence of a gluten-free diet (GFD) on microbiota changes in coeliac disease (CD) patients was described. Even though the GFD was not able to fully restore commensal microorganism abundance, the treatment was associated with the greater abundance of selected beneficial bacteria (i.a. *Bacteroides*, *Firmicutes*) and lower presence of pathogenic bacteria associated with worsening of CD symptoms (i.a. *Neisseria*, *Proteobacteria*). A similar gut microbiome structure to healthy individuals was observed in patients treated with GFD in comparison to patients consuming gluten. However, only strict adherence to GFD is required to obtain potential beneficial effects and in a long-term perspective and daily use seems to be complicated for patients [5].

I believe that this Special Issue will give readers and professionals valuable insight into the role of microbiota modulation in both health and disease conditions.
